# Microscopes and Mass Spectrometers

**DOI:** 10.4172/jpb.S10-e001

**Published:** 2016-02-15

**Authors:** Bradley Shields, Sara C Shalin, Alan J Tackett

**Affiliations:** 1 Department of Biochemistry and Molecular Biology, University of Arkansas for Medical Sciences, 4301 West Markham Street, Little Rock, Arkansas 72205, USA; 2 Department of Pathology, University of Arkansas for Medical Sciences, 4301 West Markham Street, Little Rock, Arkansas 72205, USA

## Abstract

Proteomics is a relatively young discipline while pathology is one of the oldest forms of scientific inquiry. These two fields have different methods and aims, but have many areas of overlap and shared interests. Cultivation of synergistic projects between physicians who study static images of disease and biologists who study the dynamic environment that produces disease states will help further biomedical research providing new diagnostic, prognostic, and therapeutic approaches. Here, a pathologist and a proteomic scientist share their views on recent collaborations among the fields.

## Pathologist

Pathology represents the study of disease. As a medical specialty, the field of anatomic pathology is concerned with the diagnosis of disease, both neoplastic and inflammatory. Traditionally, pathologists have relied on formalin-fixed, paraffin-embedded (FFPE) pieces of tissue, cut at 3–5 micron sections, and stained with hematoxylin and eosin to make a diagnosis. From these pink and blue slides, tumor patterns are analyzed to designate the cell of origin, inflammatory infiltrates are evaluated to find a cause, and cytologic features are examined to render a diagnosis of benign or malignant, normal or disease. As molecular biology yields insight into the genetics and protein expression patterns in tissues and tumors, these techniques have been adapted for clinical usage. The use of protein immunohistochemistry on FFPE tissue has become a widespread adjunct to diagnosis, as the proteins expressed in a cell may be a clue to the derivation of a tumor and infectious organisms may be identified. More recently, the utilization of immunohistochemistry has focused on biomarker identification/evaluation, with certain protein expression patterns predicting response to certain biologic therapies. Examples of these include Her-2-neu in breast cancers [1–3] or stomach cancers [4], c-kit expression in gastrointestinal stromal tumors [5,6], and V600E mutated melanomas [7,8].

### How does Proteomics Help?

However, the pathogenesis of normal versus disease states and tumor biology is still being explored. What is clear is that the expression of genes within a cell is only the tip of the proverbial iceberg. The field of proteomics has allowed for large scale detection of protein expression within organs, tissues, and tumors. Moreover, it is leading to a more nuanced understanding of the role of post-translational modification of proteins, the dynamic nature of such modifications, protein-protein interactions, and the rich diversity of players in the transformation of a cell from benign to malignant [9]. For pathologists and those interested in pathobiology, the ability to successfully extract and identify proteins from clinical tissues represents a frontier to be explored. Innumerable normal and diseased tissues are stored in the formalin-fixed, paraffin-embedded tissue blocks of pathology archives, and recent advances in protocols allow the successful extraction of proteomic data from these tissues [10,11]. As clinical pathology strives toward “precision-based” and “personalized” medicine in the treatment of patients, an understanding of the individual drivers of tumor biology and the opportunities for targeting out of control signaling pathways becomes more imperative. Identification of particular epigenetic modifications can now predict responsiveness to therapies, while at the same time the proteins responsible for performing these modifications can be targeted as therapeutic agents [12]. For example, histone deacetylase inhibitors are emerging as therapies for advanced cases of cutaneous T-cell lymphoma [13]. These clinical advances are due to the comprehensive research being done in proteomics and will be facilitated by continued and expanded use of FFPE samples and other clinical biospecimens.

### Examples

The manuscripts in this special volume regarding the applications of proteomics in pathology each address a different aspect of the questions left to be answered. The article by Holland and Ohlendieck reviews the potential benefits of routine proteomic analysis of skeletal muscle samples from patients with muscular dystrophy to better derive indicators of disease pathogenesis and severity. The authors emphasize the complexity of skeletal muscle and discuss the hurdles that must be overcome to successfully move the field forward, including issues of protein extraction technique, subcellular localization of various protein fractions, and identification of low abundance proteins [14]. Though written with a focus on neuromuscular disease, the challenges and complexities outlined are not unique to their chosen organ system. Samorodnitsky and colleagues ask the question whether elevated expression levels of DNA methyltransferase 1 (DMNT1) alone seen in many cancer cells is sufficient to account for the increased methylation of tumor suppressor genes that results in their transcriptional silencing or whether DMNT1 is actually more efficient at methylating DNA in tumor cells [15]. By studying neoplastic lymphocytes from patients with chronic lymphocytic leukemia and comparing them to normal lymphocytes and granulocytes, the authors found that methylation levels are not directly proportional to the increased expression of DMNT1, but that the enzyme actually binds to a subset of genes with greater cooperativity, enhancing the efficiency with which these genes are methylated and, effectively, silenced [15]. The computational analysis utilized in this manuscript suggests selective and dynamic epigenetic modifications may drive cancer progression. Haun and colleagues report successful isolation and identification of upregulated cell-surface glycoproteins using cell-surface capture mass spectrometry from cell lines of pancreatic cancer, with subsequent confirmation of protein expression of one of those proteins-CD109-in FFPE tissues from patients with pancreatic adenocarcinoma [16]. This manuscript illustrates an ideal application for FFPE clinical samples in the validation of data obtained from proteomic analyses of cell lines and other *in vitro* systems. Wong and Cox perform a systematic review of twelve proteomic studies of pre-eclampsia, a disease of aberrant placenta development, leading to significant potential maternal and fetal complications. By cross-comparing the different studies, in which both maternal serum and placental tissue was analyzed for global changes in protein expression patterns, the authors pooled the data to extract reproducible results [17]. Using this method, they found 53 proteins which were differentially expressed in at least two of the twelve studies and two specific proteins with early dysregulation that they hypothesize could serve as biomarkers to detect the disease early in pregnancy, and thereby minimize damage to mother and baby [17]. Such systematic reviews provide yet another mechanism to filter the massive amounts of data generated by proteomic analysis and focus our attention to pathways and proteins that are being found in similarly conducted experiments.

### Conclusion

The field of proteomics has produced vast amounts of data regarding the protein expression repertoire of many cell types and disease states. The continued collaboration of pathologists and molecular biologists will ensure that signaling pathways and epigenetic signatures characterized in cell culture and animal model systems can be validated in patient tissue samples. These experiments will allow for continued biomarker discovery and validation, will provide a platform for drug discovery, and ultimately will achieve a richer understanding of human biology and pathology.

## Proteomic Scientist

Proteomics is the study of the protein complement of the genome. Proteomics emerged as it became increasingly apparent that DNA sequences only provide what information is coded for within a cell and do not provide insight into how a cell might express these genes to function in a dynamic environment. In other words, the proteome is the applied genome, and is altered by both genome-directed events (protein translation) and non-genome-directed events (post-translational modifications and interactions with other cellular molecules) that are in constant flux depending on the real-time physiology of the cell and its environment [18]. The backbone of proteomic experimental methods includes separating proteins from a sample using methods such as gel electrophoresis and subsequently performing mass spectrometry on the resolved proteins, which allows for their identification and quantification. Proteomics technology has grown in pace with the information age, and technologies just a few years old are considered vastly outdated. Proteomics laboratories study many topics including: protein interactions, protein function, drug design, gene expression, and biomarker discovery for diagnosis and treatment of disease.

### How can Pathologists Help?

There is a virtual “sea” of data being generated by proteomics groups. Investigators in this fast-growing field are often seeking new questions—and pathologists are playing an emerging role in assisting and guiding proteomic investigations. A hundred years ago, pathologists, armed with their microscopes, were responsible for describing the world of the “mirco,” processes and appearance of cells in disease states, hidden from the naked eye. Now, interestingly, pathologists are routinely called upon to describe and demarcate tissue biopsies containing tens of millions of cells—a “gross” area that helps to outline candidate cells for proteomic study. Perhaps this relationship is analogous to the collaboration between geologists (the pathologist) who explains mountain range formation to the soil scientist (the proteomic basic scientist) who is studying a sample of soil in the canyon floor. Furthermore, the perspective that pathologists hold is a valuable one—they look at true normal and diseased human cells regularly. Cell lines and animal models are both effective and essential, but they do not duplicate the exact cellular state and environment of a human disease, which explains the calls for validation of studies in human tissue samples. Pathologists’ indefatigable efforts closely examining tissues of all types and states singularly equip them to assist in the generation of hypotheses in the area of proteomics. Additionally, the formalin-fixed, paraffin-embedded (FFPE) tissue blocks stored in pathology archives can serve as sources of samples in sufficient quantities to allow for identification and subsequent validation of biomarkers and drug targets. Once validated, these new biomarkers can be given back to pathologists in the form of protein immunohistochemistry targets, which can provide new diagnostic and prognostic information, as well as aid in selection of appropriate therapies.

### Example

One example of this type of synergistic relationship between pathologists and proteomic investigators concerns the study of melanoma skin cancer. Melanomas can often be distinguished from benign nevi by visual characteristics (e.g., asymmetries in shape, irregular borders, uneven color, a diameter >6 mm, and evolving characteristics) and can easily be biopsied and removed by surgical excision [19]. However upon metastasis, patient survival plummets as metastatic melanoma cells are largely unresponsive to current treatments. Understanding the molecular pathways activated in metastatic melanoma relative to early-stage disease could provide targets for new therapeutic development. Along this line of reasoning, Byrum and colleagues recently worked with pathologists to analyze a variety of benign nevi, early-stage melanomas and metastatic melanomas with high resolution proteomics. Novel and hallmark pathways were identified that provided insight into molecular mechanism and potential targets for drug development [10]. The collaboration between the pathology and proteomic groups was critical to the success of these studies.

Recently, immunotherapy has emerged as an important modality in the treatment of patients with advanced melanoma. Immune system checkpoints are co-stimulatory and co-inhibitory signals, which function to produce an immune response commensurate with the level of threat to the body. Blocking inhibitory checkpoints can be used to amplify immune system activity against tumors. The monoclonal antibodies ipilimumab (anti-CTLA4), pembrolizumab, and nivolumab (both anti-PD1) have produced an alluring hope among clinicians and patients for the treatment of metastatic melanoma. Response rates for monotherapy with immune checkpoint inhibitors have been modest (10–30%), but when patients do respond, it is often in a durable and lasting way [20]. However, there is currently no predictive model for who will respond to these therapies. Proteomics promises to play a central role in understanding which patients will respond to immunotherapies and why, which again will rely on interactions with pathologists.

### Conclusion

If you are an investigator who uses proteomics for biomedical studies—it may be beneficial to form collaborations with a pathologist. Proteomic laboratories and proteomic core facilities are often associated with Pathology departments so collaborations may already be in place at your institution. Additionally, well-documented protocols describe FFPE tissue extraction for proteomic studies, and pathologists are often the gatekeepers of these archives. As mentioned above, pathologists can be sources of fascinating ideas for research aims, but often, due to clinical responsibilities, do not have time or resources to explore them on their own. In summary, renewed application of histopathological analysis, when paired with molecular studies, may assist in the creation of more objective diagnostic and therapeutic strategies. The rapid technological advances in systems biology driven by proteomic basic scientists, coupled with insightful aims and guidance for investigations provided by pathologists, can produce the stuff of which great science is made.
